# Correction: Isolation and characterization marine bacteria capable of degrading lignin-derived compounds

**DOI:** 10.1371/journal.pone.0259490

**Published:** 2021-10-28

**Authors:** Peng Lu, Weinan Wang, Guangxi Zhang, Wen Li, Anjie Jiang, Mengjiao Cao, Xiaoyan Zhang, Ke Xing, Xue Peng, Bo Yuan, Zhaozhong Feng

An additional affiliation is missing for the first author. Peng Lu is also affiliated with School of Life Sciences, Jiangsu Normal University, Xuzhou, China.
