# Supplementary figures and images for: Isolation and characterization marine bacteria capable of degrading lignin-derived compounds
Source: PLoS One. 2020 Oct 7;15(10):e0240187. doi: 10.1371/journal.pone.0240187 (PMC7540876; doi:10.1371/journal.pone.0240187)

**S1 Fig. Electrophoresis image of 16S rDNA PCR products**


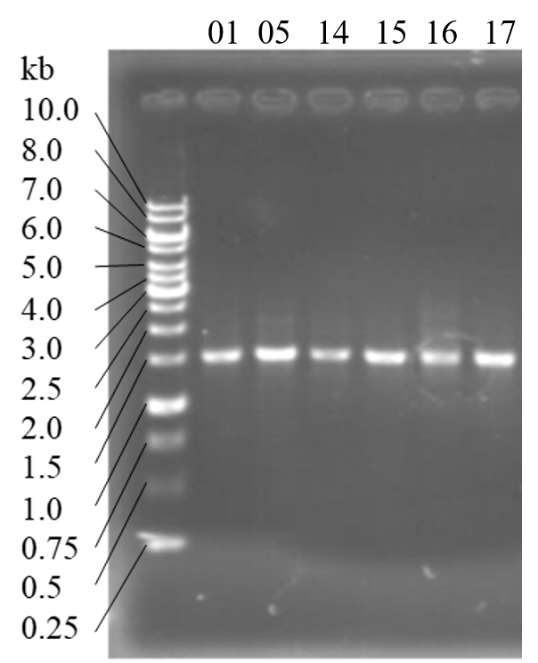

Supplement: S1 Fig — (DOCX) [file pone.0240187.s001.docx]
