# Supplementary material for: Isolation and characterization marine bacteria capable of degrading lignin-derived compounds
Source: PLoS One. 2020 Oct 7;15(10):e0240187. doi: 10.1371/journal.pone.0240187 (PMC7540876; doi:10.1371/journal.pone.0240187)
Supplement: Attachment — Submitted filename: Response to Reviewers.docx [file pone.0240187.s002.docx]

Dear editors and reviewers:

Thank you for your letter and the reviewers’ comments on our manuscript entitled " Isolation and Characterization Marine Bacteria Capable of Degrading Lignin-derived Compounds" (PONE-D-19-35662). Those comments are very helpful for revising and improving our paper, as well as the important guiding significance to other research. We have studied the comments carefully and made corrections which we hope meet with approval. The main corrections are in the manuscript and the responds to the reviewers’ comments are as follows.

Replies to the editors’ comments:

1. Please ensure that your manuscript meets PLOS ONE's style requirements, including those for file naming.

Response：Thank you for your suggestions and we have reorganized the manuscript to meet PLOS ONE's style requirements.

2. In your Methods section, please provide additional information regarding the permits you obtained for the work. Please ensure you have included the full name of the authority that approved the field site access and, if no permits were required, a brief statement explaining why.

Response：Thank you for your valuable comments. All experiments in the Methods were conducted according to protocols approved by Jiangsu Normal University and Anhui Normal University and completed by researchers of the manuscript. No additional authorization information is required for this work.

3. Please ensure that you refer to Figure 4 in your text as, if accepted, production will need this reference to link the reader to the figure.

Response：Fig 4 has been cited and all figures have been reordered and checked.

4. Please include captions for your Supporting Information files at the end of your manuscript, and update any in-text citations to match accordingly.

Response：Captions of Supporting Information has been added and rematched.

Replies to the reviewers’ comments:

1. Remove the word "Isolation" from the list of keywords.

Response：The word has been removed.

2. Revised sentence as "organic matter into the oceans" at Line No. 21.

Response：The sentence has been modified.

3. It should be "sediments" instead of "ediment" at Line No. 93.

Response：The word has been modified.

4. It should be revised as "East China Sea in Zhoushan, Zhejiang (30°58′20.71″N, 122° 95 41′22.10″E), People's Republic of China" at Line No. 94-95.

Response：The full name of China has been added, which is very important.

5. The sentence should be started with "The depth of sediments" instead of "Depth" at Line No. 95.

Response：We have modified it according to your suggestion.

6. Revised this sentence as "Further, the sediments samples were placed in sealed plastic boxes surrounded with ice and brought to the laboratory within 24h" instead of "Placed in sealed plastic boxes surrounded with ice, the samples were taken to the laboratory within 24h" at Line No. 95.

Response：We have modified it according to your suggestion.

7. Kindly mention the a tentative purity of the Chinese chemicals within bracket at Line No. 101.

Response：The purity of all chemicals has been added in the method. Thank you for your suggestion.

8. Kindly check the presence of reference both in the text as well as in the reference section.

Response：References have been checked and re-marked.

9. Kindly also incorporate the HP-LC and GC-MS chromatogram in the manuscript.

Response：Thanks for your kindly and profssional comment. In this manuscript, Fig 5 shows the analysis of FA degradation by HPLC. We used SIM (selective reaction monitoring) mode to determinated the content variation of vanillic acid, vanillin, vanillate, protocatechuate, and fumarate. In this method, five referenced ion fragments information were monitored and determined the content through calculation of ionic strength. We have reflected upon your comment and addiion of the description of method in the manuscript.

Once again, thank you very much for your constructive comments and suggestions which would help us both in English and in depth to improve the quality of the paper.

Kind regards,

Zhaozhong Feng

E-mail: fzz2012@jsnu.edu.cn
