# Supplementary material for: Isolation and characterization marine bacteria capable of degrading lignin-derived compounds
Source: PLoS One. 2020 Oct 7;15(10):e0240187. doi: 10.1371/journal.pone.0240187 (PMC7540876; doi:10.1371/journal.pone.0240187)
Supplement: Attachment — Submitted filename: Response to Reviewers.docx [file pone.0240187.s003.docx]

Dear editors and reviewers:

Thank you for your letter and the reviewers’ comments on our manuscript entitled " Isolation and Characterization Marine Bacteria Capable of Degrading Lignin-derived Compounds" (PONE-D-19-35662). Those comments are very helpful for revising and improving our paper, as well as the important guiding significance to other research. We have studied the comments carefully and made corrections which we hope meet with approval. The main corrections are in the manuscript and the responds to the reviewers’ comments are as follows.

Replies to the reviewers’ comments:

1. Line 88 – Contribute

Response：The word has been modified.

2. Line 115 – The depth of the sediment….

Response：The sentence has been modified.

3. 136 – Vanillic acid as the sole carbon source

Response：The sentence has been modified.

4. Lines 139-140- Please clarify your methods for enrichment. It is unclear what “this process” is referring to.

Response：“This process” is referring to the enrichment technique. The enrichment technique was performed in liquid ONR7a media amended with 0.2% vanillic acid. Cultures were incubated with shaking at 25 ºC until turbid.

5. Line 156- Please refer to the specific primer (ie 27 Forward) in your PCR recipe.

Response：The reference cited in the primer has been corrected.

6. Experiments described in Lines 179-188, 217 – What were your controls? How many replicates were used?

Response：Each experiment is an independent experiment, which reflects the content of metabolites according to the time course.

7. Line 207- Please include more details about your GC-MS preparation, such as extraction protocol and if the samples were derivatized.

Response：The test samples were extracted by ethyl acetate and dried over anhydrous Na_2_SO_4_ and the solvent was allowed to evaporate at 40℃. The residual was dissolved in an equal volume of methanol to original liquid sample and analyzed by GC-MS. Determination of vanillic acid, vanillin, vanillate, protocatechuate, and fumarate through SIM (selective reaction monitoring) with referenced ion fragments information. All samples were not derivatized.

8. Line 355 – Please clarify why the table shows OD values on a time scale of up to 10 days if the text is stating that the cultures were grown for 3 days with each substrate.

Response：We have modified it and the cultures were grown for 10 days with each substrate.

Once again, thank you very much for your constructive comments and suggestions which would help us both in English and in depth to improve the quality of the paper.

Kind regards,

Zhaozhong Feng

E-mail: fzz2012@jsnu.edu.cn
